# HIC1 controls cellular- and HIV-1- gene transcription via interactions with CTIP2 and HMGA1

**DOI:** 10.1038/srep34920

**Published:** 2016-10-11

**Authors:** Valentin Le Douce, Faezeh Forouzanfar, Sebastian Eilebrecht, Benoit Van Driessche, Amina Ait-Ammar, Roxane Verdikt, Yoshihito Kurashige, Céline Marban, Virginie Gautier, Ermanno Candolfi, Arndt G. Benecke, Carine Van Lint, Olivier Rohr, Christian Schwartz

**Affiliations:** 1 University of Strasbourg, EA7292, DHPI, Institut of Parasitology and tropical pathology Strasbourg, France; 2 University of Strasbourg, IUT Louis Pasteur, Schiltigheim, France; 3 Institut des Hautes Etudes Scientifiques—Centre National de la Recherche Scientifique, 35 route de Chartres, 91440 Bures sur Yvette, France; 4 Institut Universitaire de France, Paris, France; 5 Université Libre de Bruxelles (ULB), Service of Molecular Virology, Institute for Molecular Biology and Medicine (IBMM), 12 rue des Profs Jeener et Brachet, 6041 Gosselies, Belgium; 6 Deutsches Krebsforschungszentrum, Im Neuenheimer Feld 242, Heidelberg 69120, Germany; 7 CNRS UMR 7224, Université Pierre et Marie Curie, 7 quai Saint Bernard, 75005 Paris, France; 8 UCD Centre for Research in Infectious Diseases (CRID) School of Medicine and Medical Science University College Dublin, Ireland; 9 Inserm UMR 1121 Faculté de Chirurgie Dentaire Pavillon Leriche 1, place de l’Hôpital Strasbourg, France

## Abstract

Among many cellular transcriptional regulators, Bcl11b/CTIP2 and HGMA1 have been described to control the establishment and the persistence of HIV-1 latency in microglial cells, the main viral reservoir in the brain. In this present work, we identify and characterize a transcription factor i.e. HIC1, which physically interacts with both Bcl11b/CTIP2 and HMGA1 to co-regulate specific subsets of cellular genes and the viral HIV-1 gene. Our results suggest that HIC1 represses Tat dependent HIV-1 transcription. Interestingly, this repression of Tat function is linked to HIC1 K314 acetylation status and to SIRT1 deacetylase activity. Finally, we show that HIC1 interacts and cooperates with HGMA1 to regulate Tat dependent HIV-1 transcription. Our results also suggest that HIC1 repression of Tat function happens in a TAR dependent manner and that this TAR element may serve as HIC1 reservoir at the viral promoter to facilitate HIC1/TAT interaction.

Numerous cellular proteins have been described as HIV-1 transcriptional regulators involved in HIV-1 latency^1^,^2^. Among them proteins belonging to the 7sk non coding RNA (ncRNA) which include Bcl11b/CTIP2 and HMGA1 appeared to be important in the regulation of early phase, Tat-independent, HIV-1 transcription contributing to the establishment and the persistence of HIV-1 latency^3^. Indeed it was shown that CTIP2 cooperates with LSD1 and favors the formation of a compacted inactive-chromatin (heterochromatin) and the establishment of HIV-1 latency in microglial cells, the CNS resident macrophages^4^,^5^,^6^,^7^. Associated to the cellular 7SKsnRNA, CTIP2 represses P-TEFb functions and thus P-TEFb-sensitive gene transcription. The cellular High Mobility Group AT-Hook 1 (HMGA1) has been described to act in synergy with CTIP2 by recruiting CTIP2-repressed P-TEFb on the HIV-1 promoter and thus preventing HIV-1 reactivation^4^,^5^. In addition we showed that these two proteins are recruited to cellular target promoters to regulate gene expressions^5^. Additional roles of HMGA1 and CTIP2 during HIV-1 transcription and cellular gene expression remain to be deciphered. We have previously shown that CTIP2 relocates the essential viral trans activator of HIV gene expression Tat to heterochromatin protein 1 (HP1) containing structures to repress Tat-dependent HIV-1 transcription^8^. However the precise molecular mechanisms underlying this effect are not fully understood.

HMGA1 interacts with a large variety of cellular proteins involved in the regulation of HIV-1 gene transcription including SP1 and NF-KB. Moreover, HMGA1 associates with several high and low affinity DNA binding sites within the HIV-1 promoter and interacts with the TAR region of the initiated HIV-1 RNA to modulate Tat functions. These findings also point out to additional roles of HMGA1 during HIV-1 transcription^9^,^10^.

Identifying cellular factors involved in establishment and maintenance of HIV-1 latency is crucial since it helps greatly the development of more rational therapeutically strategies. The two major strategies are

1. Reactivation of virus expression aiming at the reduction of latently infected cellular reservoirs^11^
2. Reinforcement of HIV-1 repression by targeting transcriptional steps, and thus improving cART^12^

Therapies that target cellular factors appear even more important from the point of view that some of these cellular factors operate in a synergistic manner (e.g. LSD1 and CTIP2^7^ or HMGA1 and CTIP2^5^).

Among new potential HIV-1 transcriptional regulators is the cellular protein Hypermethylated In Cancer 1 (HIC1). Indeed, it has been shown that CTIP2 and HIC1 share some cellular target genes such as the p21 and the p57kip2 genes^13^,^14^,^15^,^16^. Moreover, CTIP2 and HIC1 have been reported to associate with the repressive NurD complex^15^ suggesting that both factors take part of the same complex repressing common target genes.

HIC1 is a tumor suppressor involved in numerous cellular process including DNA damage response, cell survival, and neural development^17^,^18^,^19^. Loss of heterozygosity (LOH) of *Hic1* has been implicated in many cancers, such as medulloblastoma or leukemia and has been associated with increased malignancy and poor prognosis^20^,^21^,^22^. LOH of HIC1 can occur through hypermethylation or deletion of the 17p13.3 region, where the *Hic1* gene is located^23^. HIC1 is a transcriptional repressor divided into three main regions (Fig. 1). The first domain located in the amino-terminal part is an evolutionarily conserved protein-protein interaction motif named BTB/POZ (Broad complex, Tramtrack and Bric à brac/Pox viruses and Zinc finger)^24^,^25^. This region has an autonomous transcriptional repressive activity through its interaction with the NAD-dependant class-III histone deacetylase SIRT1^26^. The central region contains two phylogenetically conserved sequences. The GLDL^225^SKK is a conserved motif to which binds the C-terminal binding protein (CtBP). Interaction of CtBP with HIC1 confers a class-I and –II HDAC-dependant transcriptional repression activity to its later one^27^. The second motif presents a SUMOylation/acetylation switch on MK^314^HEP. Acetylation of lysine 314 by CBP/P300 has been associated to a decrease of HIC1 interaction with its co-repressor. SUMOylation on the other hand has been described to activate and potentiate HIC1-mediated transcriptional repression^15^. Acetylation and SUMOylation compete for the same lysine residue^28^. Finally, the C-terminal part of HIC1 contains five krueppel-like C_2_H_2_ zinc fingers. A cluster with the last four zinc fingers allows HIC1 to interact with a 5′C/GNGC/GGGGCAC/ACC-3′ consensus sequence in HIC1 target genes, the HIC1 responsive element (HiRe)^29^. Recently zinc fingers have been described to be implicated in the recruitment of SIRT1 promoting HIC1 lysine deacetylation followed by SUMOylation favored by HDAC4^28^,^30^.

Herein we report that HIC1 interacts physically with both CTIP2 and HMGA1 to co-regulate common panels of cellular gene. In addition, we show for the first time that the tumor suppressor HIC1 is able to promote Tat-dependent HIV-1 transcriptional silencing in microglial cells. HIC1 induces nicotinamide-sensitive HIV-1 transcription extinction by cooperating with the deacetylase SIRT1. SIRT1 is known to deacetylate the lysine 314 in the MKEHP acetylation/SUMOylation switch motif of HIC1. Interestingly a single amino-acid mutation in the MKEHP acetylation/SUMOylation switch, which mimics a constitutive acetylation of the lysine 314 is sufficient to abrogate HIC1-driven transcriptional repression. Moreover, a mutant mimicking a constitutively acetylated HIC1 loses its ability to interact with Tat. We propose that SIRT1-driven HIC1 de-acetylation is a critical step which occurs upstream of HIC1/Tat interaction. Finally, we show that HIC1 interacts and cooperates with HMGA1 to regulate Tat-dependent transcription of HIV-1 gene.

## Results

### HIC1 and CTIP2 interact and co-regulate cellular genes expression

**Figure 2:**
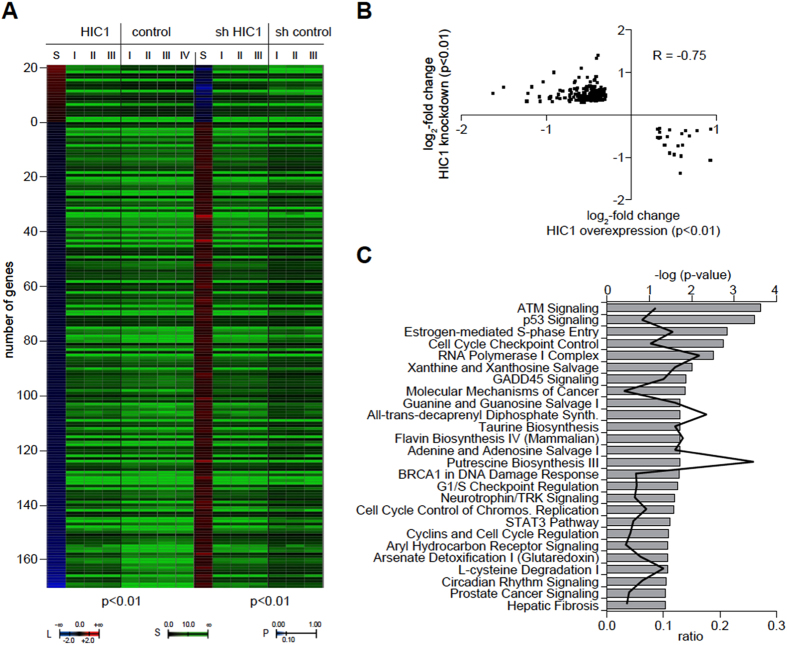
Identification of HIC1 target genes. (**A**) Heatmap of statistically significantly (p < 0.01) concordantly regulated genes upon overexpression (HIC1) and knockdown (sh HIC1) of HIC1. The number of positively regulated genes (L, red shading) and negatively regulated genes (L, blue shading) is shown together with independent biological replicate signals of each condition and control (S). Post-hoc p-values are indicated as color code (*P*). (**B**) Scatter plot of the genes shown in (**A**). The log_2_-fold change upon HIC1 overexpression is plotted against the x-axis, the log_2_-fold change upon HIC1 knockdown is plotted against the y-axis. The Pearson correlation coefficient is indicated (R). (**C**) Gene ontology pathway enrichments based on the genes shown in (**A**). Pathways significantly (p-values as indicated) enriched for HIC1 target genes are listed and the ratio is indicated.

We first took advantage of global gene expression profiling to identify and compare HIC1 and CTIP2 target genes in microglial cells. For this purpose, HIC1 and CTIP2 were either knocked down or overexpressed in microglial cells followed by transcriptome analyses using microarray technology. We defined HIC1 target genes as genes that are concomitantly regulated in statistically significant manner (p < 0.01) upon either knockdown or overexpression of HIC1 (Fig. 2A). Among the identified HIC1 targets, 21 (11%) genes were down-regulated and 171 (89%) were up-regulated upon HIC1 knockdown. The scatter plot comparing the log_2_-fold changes in the HIC1 overexpression profile with that of the HIC1 knockdown profile reveals a robust negative Pearson correlation (R = −0.75) indicating that the majority of the identified HIC1 target genes are directly regulated (Fig. 2B). As expected, pathway enrichment analyses based on these HIC1 target genes show that the ATM DNA damage response pathway as well as the apoptosis- and cell cycle control-related pathways are among the most significantly affected pathways (Fig. 2C). We have included the gene lists of cellular targets regulated by HIC1 and CTIP2 (supplemental table 1) in order to specify their identity.

**Figure 3:**
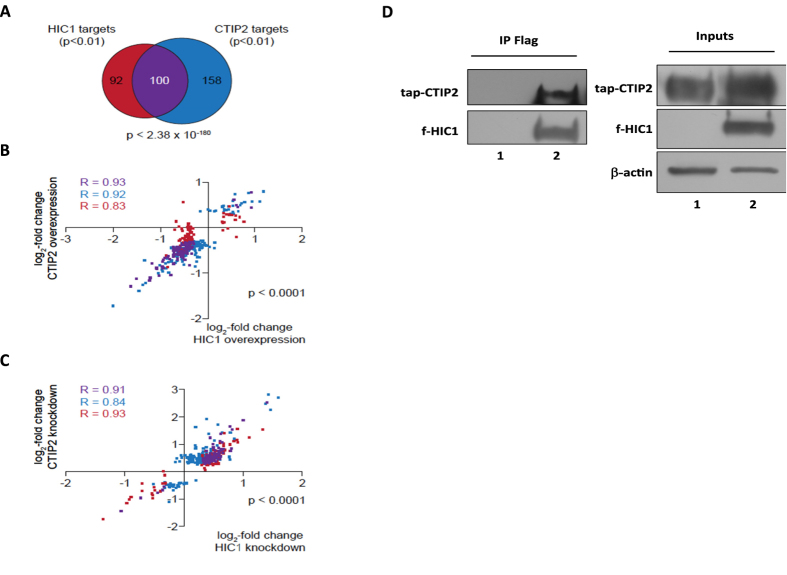
HIC1 and CTIP2 cooperate in gene expression regulation. (**A**) Venn-diagram of HIC1 and CTIP2 target genes. HIC1 target genes (red) have been identified as indicated in Fig. 1(A) and CTIP2 target genes (blue) have been defined as genes statistically significantly (p < 0.01) concordantly regulated genes upon overexpression and knockdown of CTIP2. The common subset is shown in purple. The p-value (hypergeometric distribution) for chance occurrence of this overlap is indicated. (**B**) Scatter plot of the genes shown in (**A**). The log_2_-fold change upon HIC1 overexpression is plotted against the x-axis, the log_2_-fold change upon CTIP2 overexpression is plotted against the y-axis. The Pearson correlation coefficient for each gene set is indicated (R). (**C**) as in (**B**), but comparing the log_2_-fold changes in gene expression upon HIC1 knockdown with the knockdown of CTIP2. (**D**) CTIP2 and HIC1 interact physically: HEK293T cells were co-transfected with the pNTAP-CTIP2 expression vectors (lane 1 and 2) and the pCDNA3-Flag-HIC1 expression vector (lane 2) or the control pCDNA3-Flag vector (lane 1). Complexes immunoprecipitated with the anti-Flag antibodies were immunodetected for the presence of tap-CTIP2 and Flag-HIC1 proteins by Western blot as indicated.

As in case of HIC1 we defined direct CTIP2 targets as genes that are concomitantly up or down-regulated in a statistically significant manner (p < 0.01) in CTIP2 overexpression and knockdown experiments^5^. Upon CTIP2 knock-down, 31 genes (12%) were down-regulated and 227 genes (88%) were up-regulated. The comparison of HIC1 and CTIP2 targets reveals a statistically significant (p < 2.38 × 10^−180^) common subset of 100 genes (Fig. 3A). Scatter plot representations of both gene sets as well as the common subset comparing both HIC1 and CTIP2 overexpression (Fig. 3B) and HIC1 and CTIP2 knockdown (Fig. 3C) reveal a strong positive correlation between both conditions. Taken together these results suggest that HIC1 and CTIP2 cooperate to regulate cellular genes. To determine if this functional cooperation could result from physical association, we performed co-immunoprecipitation experiments. As shown in Fig. 3D, HIC1 associated physically with CTIP2 suggesting that the observed functional cooperation may result from physical interactions between the two factors.

### HIC1 inhibits HIV-1 gene transcription and the viral expression in microglial cells

**Figure 4:**
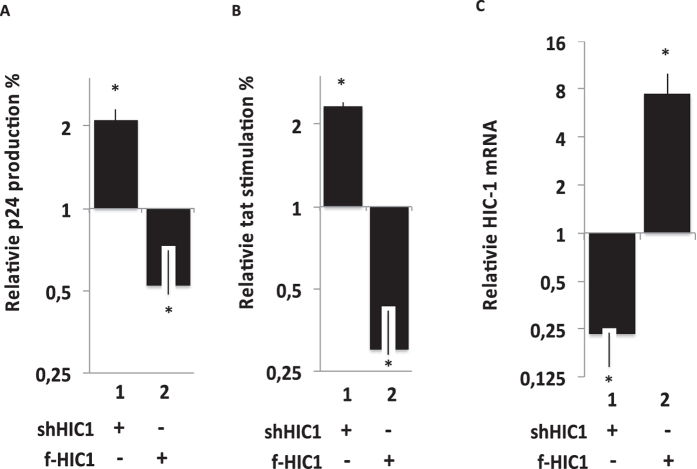
Effect of HIC1 over-expression or knock-down on HIV-1 in microglial cells. Microglial cells were transfected either with the pNL-4.3 provirus (**A**) or the episomal pLTR-luciferase reporter (**B**) and the indicated plasmids. 48-hours later, supernatants were harvested (**A**) or cells were lysed (**B**). Viral p24 concentrations were titrated in supernatant and normalized relatively to their appropriate empty vector control (**A**). Cell lysates were subjected to luciferase assay, normalized with the renilla luciferase system and expressed as relative Tat stimulation with their respective control (**B**). Nuclear cell extracts were obtained after 48-hours transfection of the indicated vectors and subjected to western-blot experiments to detect endogenous knock down and over-expressed HIC1 (respectively lane 1,2 and 3). As a control we checked the relative HIC1 mRNA following a HIC1 knock down (shHIC1) or following overexpression of flag-HIC1 (f-HIC1) (**C**).

We have previously shown that CTIP2 favors the establishment and the persistence of HIV-1 latency in microglial cells, the central nervous system major reservoirs of the virus^31^. To decipher the role of HIC1 on HIV-1 infection, microglial cells expressing a NL-4.3 provirus were subjected to HIC1 over-expression or HIC1 knock-down (Fig. 4A). Since knocking-down HIC1 promoted an increase of the virus expression, over-expressing HIC1 repressed HIV-1 p24 production. As a control, overexpression and knock-down efficiencies were assessed by RT-qPCR (Fig. 4C). These results argue for a repressive function of HIC1 on HIV-1 expression in microglial cells. Since HIC1 is known as a repressor of gene transcription, we next focused on HIC1 functions on HIV-1 gene transcription in microglial cells. As shown Fig. 4B, knocking-down HIC1 favored Tat-mediated stimulation of the HIV-1 gene promoter. As expected, overexpression of HIC1 in microglial cells repressed Tat activity (Fig. 4B). Of note, the knock-down and the overexpression of HIC1 had no significant effect on HIV-1 gene transcription in the absence of Tat (data not shown). These results suggest that HIC1 represses HIV-1 expression by counteracting Tat function at the transcription step of the viral life cycle.

### HIC1 colocalizes and interacts with Tat in microglial cells nuclei

**Figure 5:**
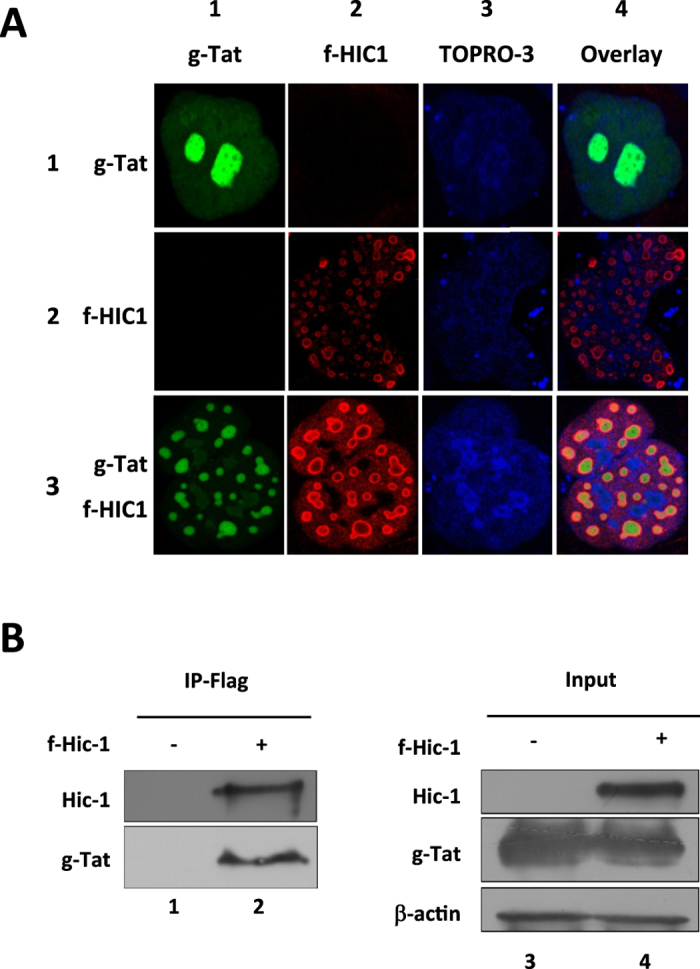
HIC1 relocates TAT in sub-nuclear ball like structures in which they interact. (**A**) Microglial cells were transfected with pGFP-TAT (row 1), pFlag-HIC1 (row 2) or both (row 3). 48-hours post-transfection cells were fixed and stained with TOPRO-3 (column 3) to detect the nucleus. Flag-HIC1 was detected by incubating cells with an anti-flag antibody and immunostained with a cyanine 3-labelled secondary antibody (column 2). Localization of Flag-HIC1 and GFP-TAT has been acquired by confocal microscopy. (**B**) HEK293T cells were transfected with pGFP-TAT alone or in combination with pFlag-HIC1. 48-hours later cells were lysed and nuclear extracts have been subjected to immunoprecipitation with an anti-Flag tag antibody. TAT and HIC1 were detected by western blot with respectively anti-TAT and anti-Flag antibodies.

Since HIC1 counteracts Tat function, we next investigated on the locations and the interactions between the cellular and the viral transcription factors. First we looked for the localization of the two proteins by confocal microscopy in microglial cells (Fig. 5A). Both proteins displayed a nuclear localization when expressed alone (Fig. 5A lane 1 and 2, column 3). However, Tat was expressed in the nucleoplasm and the nucleoli (Fig. 5A lane 1 column 1), while HIC1 was found in the outskirts of nuclear ball-like structures (Fig. 5A lane 2 column 2). Interestingly, upon co-expression, Tat relocates and co-localizes with HIC1 in the HIC1-induced ball-like structures (Fig. 5A lane 1 vs lane 3 columns 1 and 4). Since HIC1 relocates and co-localizes with Tat, we investigated whether both proteins could physically interact. We performed immunoprecipitation experiments on nuclear extract from cell expressing Flag-HIC1 and GFP-Tat. As shown in Fig. 5B, HIC1 physically associated with Tat suggesting that HIC1-mediated repression of Tat function depends on the physical association of the two proteins.

### HIC1 functionally interacts with Tat and Sirt1 to control HIV-1 gene transcription

**Figure 6:**
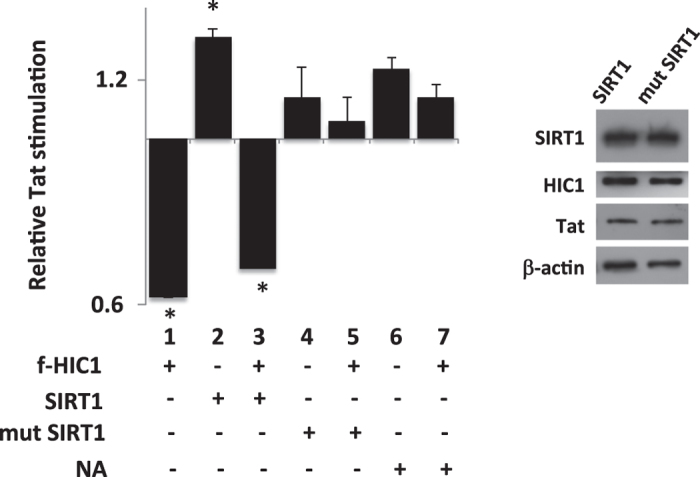
HIC1-mediated TAT-dependent HIV-1 transcription repression process requires SIRT1 deacetylase activity. Microglial cells were transfected with pLTR-Luc and the indicated expression vector. Relative Tat stimulation in the absence (lanes 2, 4 and 6) or in the presence (lanes 1, 3, 5 and 7) of flag_HIC1was studied in absence (lanes 1, 4, 5 and 6) or presence of over-expression of either wild-type SIRT1 (lanes 2 and 3) or H^363^Y SIRT1 mutant (lanes 4 and 5). 24-hours after transfection, cells were either untreated (lanes 1,2, 3, 4 and 5) or incubated with 10 mM nicotinamide (NA) (lanes 6 and 7). 48-hours post-transfection, cells were lysed and luciferase activity measured. Values were normalized using renilla luciferase system. Empty vector transfection of each condition has been set to one and other transfection points were normalized accordingly.

Next we hypothesized that SIRT1, the mammalian homolog of Sir2 enzymes, may also be involved in HIC1 mediated regulations. Indeed, this class-III NAD+ dependent deacetylase has been described as a co-repressor of HIC1 and a co-activator of Tat^26^,^32^ and Fig. 6 (lane 2). To investigate this point, we evaluated the impact of SIRT1 on HIC1-mediated inhibition of Tat function (Fig. 6). Overexpression of WT-SIRT1 did not drastically modify HIC1 repression of Tat transactivation (Fig. 6: 40% of Tat inhibition lane 1 vs 50% in lane 3). However, overexpression of SIRT1 H^363^Y mutant completely abrogated HIC1 mediated repression (Fig. 6: lane 5 vs row 1). The single amino-acid mutated H^363^Y SIRT1 variant is catalytically inactive^33^. These results together suggest that the mutated form of SIRT1 competes with the endogenous one to modulate HIC1 function. In addition, our results suggest that the deacetylase function of SIRT1 is involved in HIC1-mediated regulation of Tat function. To further validate the role of SIRT1 deacetylase activity on HIC1–mediated repression, we used nicotinamide (NA), the most potent inhibitor of SIRT1^34^, and evaluated the impact of its treatment on HIC1 function (Fig. 6). NA treatments abolished HIC1-mediated repression of Tat function (Fig. 6 lane 7 vs lane 1), confirming the need of the catalytic activity of SIRT1 in the regulation of HIV-1 promoter by HIC1.

### HIC1 Lysine 314 is crucial for Tat association and HIC1-mediated repression of the HIV-1 gene transcription

The central region of HIC1 contains a MK^314^HEP conserved sequence, in which the lysine 314 (K^314^) acts as an acetylation/SUMOylation switch. Acetylation and deacetylation of HIC1 K^314^ is respectively under control of the cellular acetyl-transferase CBP/P300 and SIRT1^28^. SIRT1 binds the krueppel-like C_2_H_2_ zinc fingers in the carboxy-terminal end of HIC1 and promotes deacetylation of K^314^ by its non-histone protein deacetylase activity^30^. Meanwhile, SIRT1 also recruits HDAC4, known as an E3 ligase of the SUMO pathway^35^, which facilitates the SUMOylation of K^314^. SUMOylation of HIC1 has previously been associated with recruitment of the co-repressor NuRD through the interaction of HIC1 and the subunit MTA1 of NuRD^15^. Single amino-acid modifications in the MK^314^HE^316^P sequence modify the post-translational pattern of HIC1. Thus mutation of the glutamic acid 316 (E^316^A) disrupts the SUMOylation ψKxEx motif and abolishes HIC1 SUMOylation. The K^314^ is the target of both SUMOylation and acetylation post-translationnal marks. When K^314^ is mutated to an arginine (K314R), HIC1 can neither be acetylated nor SUMOylated^28^. However mutation of K^314^ to glutamine (K314Q) mimics a constitutively acetylated HIC1, probably because the side chain of glutamine is similar in charge and structure to an acetylated lysine.

**Figure 7:**
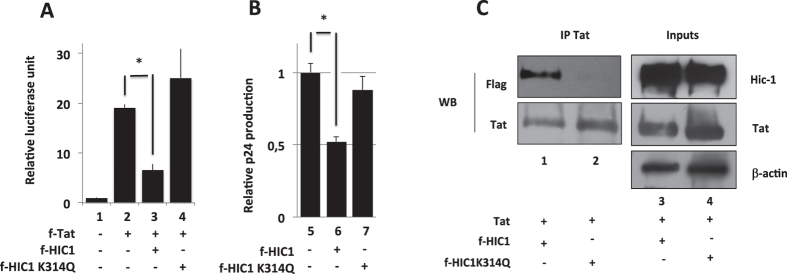
Acetylation of lysine 314 is detrimental to HIC1 inhibitory activity and disrupts HIC1/TAT association. HIC1 central domain contains a MK^314^HEP conserved sequence, in which the lysine 314 (K^314^) acts as an acetylation/SUMOylation switch (Fig. 1). SIRT1 interacts with the HIC1 5 krueppel-like zinc fingers and deacetylates K^314^. SIRT1 recruits also HDAC4 which facilitate SUMOylation of K^314^ (Fig. 1). Microglial cells were co-transfected with the indicated constructs and either episomal pLTR-Luc (**A**) or pNL-4.3 viral genome (**B**). 48-hours post-transfection, cells were lysed and subjected to luciferase assay, while normalized with renilla luciferase system (**A**) or supernatants were harvested and p24 concentration titrated (**B**). Values are normalized relatively to basal level corresponding to empty vector (**A,B**), lane1. HEK293T cells were transfected with pFlag-HIC1 wild-type (**C**), lane 3 or the pFlag-HIC1 mutant K^314^Q (**C**), lane 4 in presence of TAT. Nuclear cell extracts were obtained after 48-hours transfection of the indicated vectors and subjected to western-blot experiments with anti-flag and anti-TAT antibodies (**C**), Inputs lanes 3 and 4. Cells were lysed and nuclear extracts have been subjected to immunoprecipitation with an anti-TAT antibody (**C**). Co-immunoprecipitations of Flagged HIC1 wild type and K^314^Q mutant with TAT were compared by western-blot with respectively anti-flag and anti-TAT antibodies (**C**) IP Tat lanes 1 and 2).

Our results suggest that SIRT1 cooperates with HIC1 to repress HIV-1 transcription. Since SIRT1 deacetylates and facilitates SUMOylation of HIC1, we investigated whether these specific post-translational modifications could be involved in the repression process. Along with this goal, we investigated the effects of mutated HIC1 proteins on HIV-1 gene transcription and on the viral expression. As shown in Fig. 7A,B, WT HIC1,effectively repressed Tat-mediated transactivation of the HIV-1 promoter (Fig. 7A lane 3 vs lane 2) and p24 production (Fig. 7B lane 6 vs lane 5). However, the mutation of the Lysine 314 to a glutamine (K^314^Q) (Fig. 7A lane 4) but not the E^316^A and K314R mutations (data not shown) abrogated HIC1 repressive function. As attested by expression profile, the mutation K^314^Q did not significantly impact HIC1 expression (Fig. 7C). Hence the impacts of the HIC1 mutant K^314^Q was similar in the context of NL4.3 expressing microglial cells. Indeed, the K314Q mutation abrogated HIC1-mediated repression of the HIV-1 expression (Fig. 7B lane 7 versus 6). Since we have reported that HIC1 physically interacts with Tat to repress HIV-1 gene transcription, we further compared the ability of the WT and the K314Q mutated HIC1 proteins to interact with Tat. In agreement with the results shown in figure 5, WT HIC1 but not the K314Q mutant interacted with Tat (Fig. 7C).

Taken together, our results suggest that mimicry of the acetylation mark on K^314^ (K314Q) impaired HIC1 association to Tat, which was correlated with a repressed-transcription and an impaired-expression of the HIV-1.

### The TAR region of the HIV-1 gene transcripts is involved in transcriptional repression mediated by HIC1

**Figure 8:**
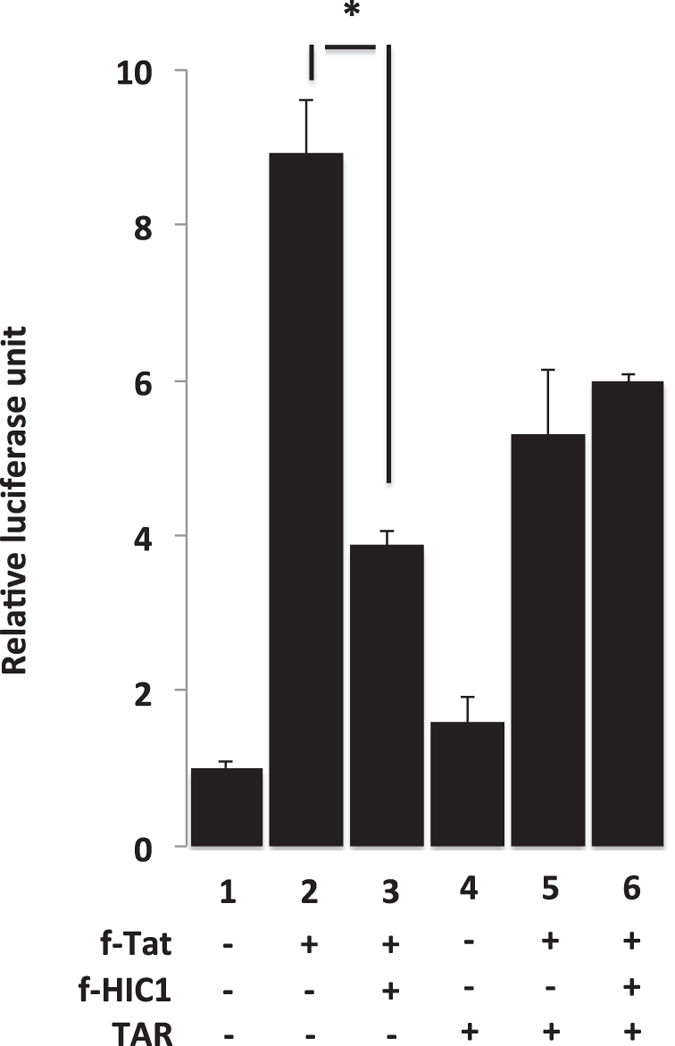
The TAR RNA is a critical element of HIC1 inhibitory activity. Microglial cells were co-transfected with pLTR-luc in presence of TAT (lanes 2 to 6) with (lanes 3 and 6) or without HIC1 (lanes 1, 2, 4 and 5). The implication of the TAR element in the HIC1-mediated HIV-1 transcription was assessed by comparing HIC1 repressive activity in absence (lanes 1, 2 and 3) or presence of free TAR RNA (lanes 4, 5 and 6). 48-hours post-transfection, cells were lysed and luciferase activity measured. Values were normalized using renilla luciferase system.

Elongation of HIV-1 gene transcripts is initiated by the binding of the viral Tat protein on the TAR region of the initiated transcripts, which in turn recruits P-TEFb and promotes RNApol II processivity. We have investigated the impact of the Tat-TAR axis on HIC1 function by evaluating the effects of exogenous TAR RNA on the HIV-1 promoter activity. Expression of exogenous TAR RNA in the absence of Tat did not significantly affect the basal activity of the HIV-1 promoter (Fig. 8 row 4 vs 1). Moreover, we observed a competition between the exogenous TAR RNA and the LTR-TAR region, which, as a consequence, repressed Tat-mediated activation (Fig. 8, row 5 vs 2). Surprisingly, exogenous TAR RNA also abrogated HIC1-mediated repression of Tat activity (Fig. 8, row 5 vs 6). These results may suggest that HIC1-mediated repression of Tat function occurs in a TAR dependent manner.

### HIC1 cooperates with HMGA1 to target gene transcriptions

**Figure 9:**
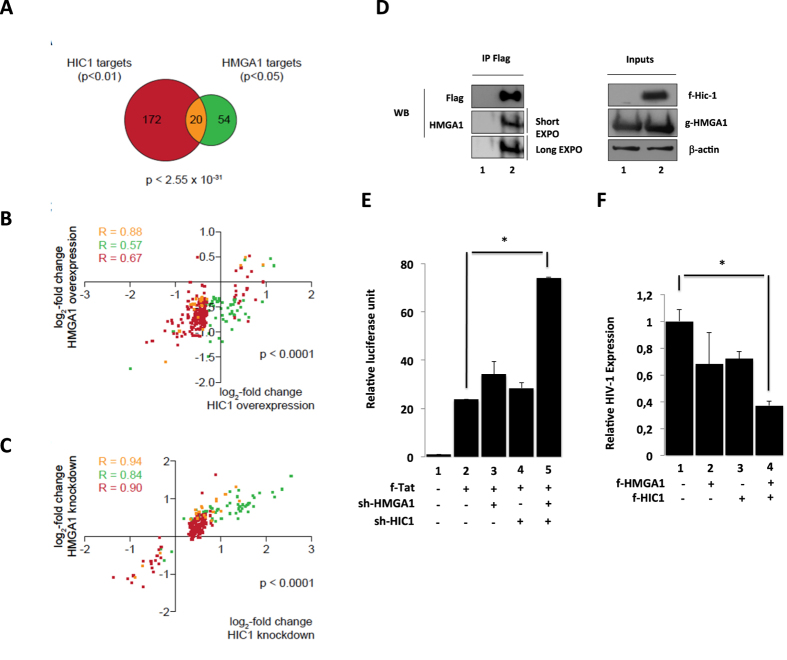
Cooperativity of HIC1 and HMGA1. (**A**) Venn-diagram of HIC1 and HMGA1 target genes. HIC1 target genes (red) have been identified as indicated in Fig. 1A and HMGA1 target genes (green) have been defined as genes statistically significantly (p < 0.05) concordantly regulated genes upon overexpression and knockdown of HMGA1. The common subset is shown in yellow. The p-value (hypergeometric distribution) for chance occurrence of this overlap is indicated. (**B**) Scatter plot of the genes shown in A. The log_2_-fold change upon HIC1 overexpression is plotted against the x-axis, the log_2_-fold change upon HMGA1 overexpression is plotted against the y-axis. The Pearson correlation coefficient for each gene set is indicated (R). (**C**) As in (**B**) but comparing the log_2_-fold changes in gene expression upon HIC1 knockdown with the knockdown of HMGA1. **(D**) HMGA1 and HIC1 interact physically: HEK293T cells were transfected with the GFP-HMGA1 expression vector and the Flag-HIC1 expression vector (lane 2) or the control pCDNA3-Flag vector (lane 1). Complexes immunoprecipitated with the anti-Flag antibodies were immunodetected for the presence of Flag- HIC1 and HMGA1 proteins by Western blot as indicated. (**E,F**) HIC1 cooperates with HMGA1 to repress HIV-1 gene transcription and viral replication: Microglial cells were transfected either with the episomal pLTR-luciferase reporter (**E**) or the pNL-4.3 provirus (**F**) and the indicated plasmids. 48-hours later, cells were lysed (**E**) or supernatants were harvested (**F**). Relative HIV expression was measured through the titration of the viral p24 concentrations in supernatant and normalized relatively to their appropriate empty vector control (**F**). Cell lysates were subjected to luciferase assay, normalized with the renilla luciferase system and expressed as relative value with their respective control (**E**).

We have previously shown that interaction of the cellular transcription factor HMGA1 with TAR modulates Tat-dependent HIV-1 gene transcription^36^. In addition, we have reported that CTIP2-associated P-TEFb complex is recruited by HMGA1 to the HIV-1 promoter and cellular target promoters^5^. In view of these observations and the above-presented results we examined the potential functional and physical interactions of HMGA1with HIC1. First we compared HIC1 and HMGA1 target gene expression profile. HMGA1 target genes have been defined as genes that are concomitantly and statistically significantly (p < 0.05) regulated upon HMGA1 knockdown and overexpression^5^. In microglial cells 6 genes (8%) are down-regulated and 68 genes (92%) are up-regulated upon HMGA1 knockdown. Comparing HIC1 targets with HMGA1 targets reveals a statistically significant (p < 2.55 × 10^−31^) common subset of 20 genes (Fig. 9A). The HIC1 and HMGA1 target genes show a strong positive correlation when comparing either HIC1 overexpression or HMGA1 overexpression (Fig. 9B) and either HIC1 knockdown or HMGA1 knockdown (Fig. 9C). Remarkably, this positive correlation is also observed for each target gene set alone, as indicated by the Pearson correlation coefficients. We have included the gene lists of cellular targets regulated by HIC1 and HMGA1 (supplemental Table 2) in order to specify their identity.

These observations support a cooperative function of HIC1 and HMGA1 in the regulation of cellular target genes in microglial cells. We performed co-immunoprecipitation experiments in order to determine whether HIC1 and HMGA1 interact in cells. As shown in Fig. 9D, HMGA1 was co- immunoprecipitated with HIC1 suggesting a physical interaction between the two proteins. We next focused on other putative functional interactions in the regulation of HIV-1 expression. We observed that a concomitant reduction of endogenous HIC1 and HMGA1 promoted a strong synergistic stimulation of HIV-1 transcription (Fig. 9E, row 5 (74 folds) vs 2 (Tat alone: 23 folds)). Of note, this effect was observed only if moderate amount of sh constructs (sh-HMGA1 and Sh-HIC1) were used. In the experiments only modest raise of Tat-mediated activation was observed when sh-HMGA1 (34 fold) or sh-HIC1 (28 fold) were co-expressed with Tat (Fig. 9E, row 3 and 4 vs 2). To reinforce these results, we performed concomitant overexpression of HIC1 and HMGA1 in HIV-1 infected cells (Fig. 9F). As previously shown, even moderate overexpression of HIC1 and HMGA1 repressed HIV-1 expression (Fig. 9F, row 2 and 3 vs 1). Moreover, concomitant HIC1 and HMGA1 overexpression promoted a cooperative repression that inhibited more than 60% of HIV-1 expression. Taken together, our results suggest that HIC1 and HMGA1 interact physically and functionally to repress HIV-1 and cellular gene expression.

## Discussion

We show in this paper that the tumor suppressor protein HIC1 interacts physically with CTIP2 and HMGA1 to regulate subsets of cellular genes. Among the cellular target genes regulated by HIC1, CTIP2 and HMGA1 are several, which have been reported to be involved in HIV-1 infection. The tudor domain containing 7 (TDRD7) gene, which is down-regulated by HIC1 and CTIP2, belongs to a group of interferon-stimulated genes, which repress HIV-1 replication^37^. The interferon-induced transcription of the dickkopf homolog 1 (DKK1) gene, an antagonist of the beta-catenin pathway which is inhibited by HIC1 and CTIP2, activates HIV-1 LTR activity^38^. Repression of the cyclin-dependent kinase inhibitor 1A, which is regulated upon HIC1- CTIP2- and HMGA1 overexpression, has been shown to impact the viral mRNA production in CD4+ T cells^39^. The regulation of these cellular target genes may support the Tat/TAR-dependent effect of HIC1, CTIP2 and HMGA1 on HIV-1 transcriptional repression. Notably, 95 out of 100 genes within the common subset of HIC1 and CTIP2 targets are repressed by the overexpression of either protein and induced by the knockdown of the corresponding protein. Also in the case of the common subset of HIC1 and HMGA1 targets, the majority (17 out of 20) are repressed upon HIC1 and HMGA1 overexpression and activated by their knockdown, respectively. Both observations support a primarily repressive cooperativity of HIC1 and CTIP2 as well as of HIC1 and HMGA1 on cellular gene expression. However, this repressive effect on cellular genes most likely involves a different mechanism as compared to the Tat/TAR-dependent HIV-1 repression, which needs to be further investigated in future studies.

We also show that HIC1 is new transcriptional inhibitor acting during the late phase of Tat-dependent HIV1 transcription. We notably demonstrated that HIC1 repressive activity requires SIRT1 deacetylase activity in microglial cells. Moreover, a single mutation in the MK^314^HEP acetylation rendering HIC1 constitutively acetylated is sufficient to abolish HIC1 inhibitory effect. Constitutively acetylated HIC1, unlike HIC1 wild type, is neither able to repress Tat-mediated HIV-1 transcription nor to interact with Tat, although the SIRT1/HIC1 interaction still remains. It seems that SIRT1/HIC1 interaction may promote deacetylation of HIC1, which facilitates HIC1/Tat interaction. We also found that HIC1 is member of at least one TAR-containing complex (data not shown). Our results suggest that the TAR element may serve as HIC1 reservoir at the viral promoter to facilitate HIC1/TAT interaction. They also suggest that HIC1 repression of Tat function occurs in a TAR dependent manner.

Finally, we show that HIC1 interacts physically and functionally with HMGA1 to repress Tat dependent HIV-1 transcription repression. Surprisingly, we could not observe a clear functional cooperation between CTIP2 and HIC1 on the control of HIV-1 replication (data not shown). Indeed we have previously shown that CTIP2 and HMGA1 acted synergistically to prevent HIV-1 reactivation^5^ and in the present paper that HMGA1 and HIC-1 inhibited Tat-dependent transcription. These results suggest us that different complexes containing CTIP2, HIC1 or HMGA1 alone or together either CTIP2 and HIC1, CTIP2 and HMGA1, and HIC1 and HMGA1 may exist to fulfill different functions. These different complexes reflect a dynamic process involved in fine-tuned regulation of both cellular and viral gene and might be related to posttranslational modification of these proteins. In favor to this hypothesis we showed that Protein Kinase C-Mediated phosphorylation of CTIP2 at Serine 2 negatively regulates its interaction with NuRD Complexes during CD4+ T-Cell activation^40^.

The precise mechanisms of the repression exerted by HIC1 alone or in association with HMGA1 is far to be elucidated and will need more investigations but we know that it is occurring in presence of Tat and is independent of P-TEFb (data not shown). HMGA1 alone is not able (Fig. 9E) or represses very faintly^36^ the late phase of the Tat dependent HIV1 transcription but requires the interaction with HIC1, which suggests a new role for HMGA1 during HIV-1 transcription. A recent study indicated that HMGA1 interacts with TAR RNA Binding Protein (TRBP)^41^. In our opinion HMGA1 could be the link between HIC1 and HIV-1 TAR RNA through interaction with TRBP and will need further investigation.

The transcription inhibition mechanism has not been elucidated yet and may involve a complex interplay between HIC1, SIRT1, HMGA1 and Tat. SIRT1 and Tat are indeed known to interact at the end of the transactivation cycle. SIRT1 removes acetylation marks of lysine 28 and 50 of Tat allowing the recycling of the viral transactivator^42^. Deciphering the sequential process occurring between HIC1, SIRT1 and Tat would allow us to understand the modalities of HIC1-mediated Tat-transactivation repression in the prevention of HIV-1 silencing.

Finally, our results have allowed the identification of new potential targets for HIV-1 reactivation i.e. SIRT1 as well as HIC1 in a “shock and kill” strategy. Such a strategy aims to at least reduce the size of the cellular reservoir which might lead to a functional cure through the association of reactivation of HIV transcription (shock) to an intensifying cART and/or novel immunological approaches for HIV-1 eradication (kill)^43^,^44^. We and others have reported the efficiency of combined treatments for reactivating quiescent/latent HIV both *in vitro, ex vivo* and *in vivo*^45^,^46^,^47^,^48^,^49^ (reviewed in ref. 50).

## Materials and Methods

### Plasmids

Most of the constructs used in our assays have been previously described: pcDNA3, pFlag-TAT^51^, pGFP-TAT^8^, pFlag- CTIP2^6^, pFlag-HIC1, pFlag-HIC1 K^314^Q, pFlag-HIC1 K^314^R, pFlag-HIC1 E^316^A^28^, the shHMGA1 and GFP-HMGA1^52^,^53^, the pFlag-SIRT1 et pFlag-SIRT1 H^363^Y^54^, the episomal pLTR-Luc^6^, pNL-4.3^55^. pshHIC1 and pshscramble control were manufactured by Suresilencing^®^ (Qiagen). For luciferase assays we used as an internal control the reporter encoding the Renilla luciferase gene (Promega).

### Cell culture

Human microglial cells^56^ and HEK293T cells were cultivated in Dulbecco’s Modified Eagle’s Medium (DMEM) supplied with 10% fetal calf serum and 100 U/ml penicillin-streptomycin.

### Luciferase Assay

Microglial cells were cultivated in 48-wells plate and transfected with fixed amount of plasmid DNA using calcium phosphate precipitation technique. Nicotinamide (NA) treatment was performed 24-hours after transfection. Cells were incubated with 10 mM NA or were mock treated with the proper diluent. 48 hours post-transfection cells were subjected to Dual-Glo^®^ Luciferase Assay System (Promega) and luciferase activity was measured. Values are representative of at least three independent experiments in at least duplicates. Basal activity which corresponds to transfection with only control vectors, were adjusted to one.

### P24 ELISA assay

Microglial cells were cultivated in 48-wells plate and transfected with 1 μg/well of plasmid DNA using calcium phosphate precipitation technique. 48 hours post-transfection cell supernatants were harvested and subjected to Innotest^®^ p24 ELISA Assay (Innogenetics). Values are representative of at least three independent experiments in at least duplicates. Basal activity which corresponds to transfection with only control vectors, were set at one.

### Co-immunoprecipitation assays

HEK293T cells were cultivated in 144 mm petri-dishes and transfected with 75 μg of plasmid DNA using calcium phosphate precipitation method. 48-hours post-transfection cells were harvested and lysed to recover nuclear proteins. Nuclear extracts (1 mg) were incubated overnight at 4 °C with M2 anti-Flag antibody (SIGMA), anti-TAT antibody (abcam) or anti-SIRT1 antibody (Millipore). Complex proteins/antibody were washed with both high-salt (500 mM NaCl) and low salt (125 mM NaCl) buffers. Complexes were studied by SDS-PAGE and Western blot.

### SDS-PAGE and Western blot analysis

SDS-PAGE was performed using standard procedures. Proteins were western-blotted with M2 anti-flag antibody (Sigma), anti-TAT antibody (abcam), anti-SIRT1 antibody (Millipore) and anti-HMGA1 (abcam). Proteins were detected using the Super Signal West Dura^®^ Chemiluminesence Detection System (Pierce biotech).

### Immunocytochemistry and confocal microscopy

Microglial cells were cultivated on glass coverslips in 24-wells plate and transfected with Jetprime^®^ (polyplus transfection) according to manufacturer protocol. 48-hours post-transfection cell were fixed with PFA 4% and permeabilized with PFA4%/triton1%. Cells were incubated in BSA blocking buffer, then incubated 1 hour with primary mouse M2 anti-Flag antibody (Sigma) followed by a 1-hour incubation with cyanine 3-labelled anti-mouse antibody. Finally, cells were incubated 30 minutes with TOPRO-3 to stained cell nucleus. Cells were washed with PBS between each step. Fluorescence was recorded by confocal microscopy using a Zeiss laser scanning microscope (model 510 invert) equipped with a Planapo oil (63x) immersion lens (numerical aperture = 1.4).

### Gene expression profiling and data analysis

Total RNA from transfected microglial cells was prepared using the RNeasy Midi Kit (Qiagen) as recommended by the manufacturer. The RNA was labelled using the Illumina^®^ TotalPrep™ RNA Amplification Kit (Ambion) according to the manufacturer instructions. For microarray analyses, hybridization and detection were performed following the protocols supplied by Illumina using the HumanHT-12 v4 Expression BeadChip Kit and an iScan system (both Illumina). The raw data were quality controlled^57^, NeoNORM normalized using k = 0.2^58^, and analyzed as outlined in^59^. For subtraction profiling we used the CDS statistical test^60^ with a positive False Discovery Rate correction where appropriate. Canonical pathway enrichment studies were performed using Ingenuity Pathway Analysis^®^ software (Ingenuity^®^ Systems) as recommended by the manufacturer. Transcriptome data were deposited in the public database MACE (http://mace.ihes.fr) using Accession Nos.: 3037572262 (CTIP2 knock-down), 2166467750 (CTIP2 overexpression), 3164892326 (HMGA1 knock-down), 2365090982 (HMGA1 overexpression), 2574019750 (HIC1 knock-down) and 3138153638 (HIC1 overexpression).

## Additional Information

**How to cite this article**: Le Douce, V. *et al.* HIC1 controls cellular- and HIV-1- gene transcription via interactions with CTIP2 and HMGA1. *Sci. Rep.* **6**, 34920; doi: 10.1038/srep34920 (2016).

## Supplementary Material

Supplementary Table 1

Supplementary Table 2

## Figures and Tables

**Figure 1:**
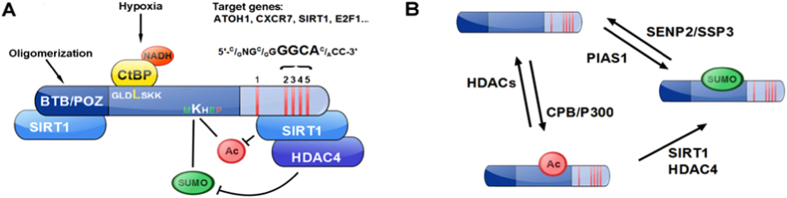
Schematic structure of HIC1 and post translational modifications. HIC1 is a transcriptional repressor divided in three main regions (**A**) (1) A BTB/POZ protein-protein interaction domain located in the amino-terminal part (2) A HIC1 central domain which contains a MK^314^HEP conserved sequence, in which the lysine 314 (K^314^) acts as an acetylation/SUMOylation switch (3) A C-terminal part which contains five krueppel-like C_2_H_2_ zinc fingers. (**B**) SIRT1 interacts with the HIC1 5 krueppel-like zinc fingers and deacetylates K^314^. SIRT1 recruits also HDAC4 which facilitate SUMOylation of K^314^.

## References

[b1] Le Douce V., Herbein G., Rohr O. & Schwartz C. Molecular mechanisms of HIV-1 persistence in the monocyte-macrophage lineage. Retrovirology 7, 32 (2010). 20380694 10.1186/1742-4690-7-32 PMC2873506

[b2] Van Lint C., Bouchat S. & Marcello A. HIV-1 transcription and latency: an update. Retrovirology 10, 67 (2013). 23803414 10.1186/1742-4690-10-67 PMC3699421

[b3] Eilebrecht S., Schwartz C. & Rohr O. Non-coding RNAs: novel players in chromatin-regulation during viral latency. Curr. Opin. Virol. 1–7, doi: 10.1016/j.coviro.2013.04.001 (2013). 23660570

[b4] Cherrier T. *et al.* CTIP2 is a negative regulator of P-TEFb. Proc. Natl. Acad. Sci. USA. 110, 12655–60 (2013). 23852730 10.1073/pnas.1220136110 PMC3732990

[b5] Eilebrecht S. *et al.* HMGA1 recruits CTIP2-repressed P-TEFb to the HIV-1 and cellular target promoters. Nucleic Acids Res. 42, 4962–71 (2014). 24623795 10.1093/nar/gku168 PMC4005653

[b6] Marban C. *et al.* Recruitment of chromatin-modifying enzymes by CTIP2 promotes HIV-1 transcriptional silencing. Embo J 26, 412–423 (2007). 17245431 10.1038/sj.emboj.7601516 PMC1783449

[b7] Le Douce V. *et al.* LSD1 cooperates with CTIP2 to promote HIV-1 transcriptional silencing. Nucleic Acids Res 1904–15 (2011). 22067449 10.1093/nar/gkr857 PMC3300010

[b8] Rohr O. *et al.* Recruitment of Tat to heterochromatin protein HP1 via interaction with CTIP2 inhibits human immunodeficiency virus type 1 replication in microglial cells. J Virol 77, 5415–5427 (2003). 12692243 10.1128/JVI.77.9.5415-5427.2003 PMC153947

[b9] Henderson A., Bunce M., Siddon N., Reeves R. & Tremethick D. J. High-mobility-group protein I can modulate binding of transcription factors to the U5 region of the human immunodeficiency virus type 1 proviral promoter. J. Virol. 74, 10523–34 (2000). 11044097 10.1128/jvi.74.22.10523-10534.2000 PMC110927

[b10] Reeves R. Molecular biology of HMGA proteins: hubs of nuclear function. Gene 277, 63–81 (2001). 11602345 10.1016/s0378-1119(01)00689-8

[b11] Le Douce V. *et al.* Achieving a cure for HIV infection: do we have reasons to be optimistic? J. Antimicrob. Chemother. 67, 1063–74 (2012). 22294645 10.1093/jac/dkr599 PMC3324423

[b12] Le Douce V. *et al.* Improving combination antiretroviral therapy by targeting HIV-1 gene transcription. Expert Opin. Ther. Targets, doi: 10.1080/14728222.2016.1198777 (2016). 27266557

[b13] Dehennaut V., Loison I., Boulay G., Van Rechem C. & Leprince D. Identification of p21 (CIP1/WAF1) as a direct target gene of HIC1 (Hypermethylated In Cancer 1). Biochem. Biophys. Res. Commun. 430, 49–53 (2013). 23178572 10.1016/j.bbrc.2012.11.045

[b14] Cherrier T. *et al.* p21(WAF1) gene promoter is epigenetically silenced by CTIP2 and SUV39H1. Oncogene 28, 3380–3389 (2009). 19581932 10.1038/onc.2009.193 PMC3438893

[b15] Van Rechem C. *et al.* Differential regulation of HIC1 target genes by CtBP and NuRD, via an acetylation/SUMOylation switch, in quiescent versus proliferating cells. Mol. Cell. Biol. 30, 4045–59 (2010). 20547755 10.1128/MCB.00582-09 PMC2916445

[b16] Topark-Ngarm A. *et al.* CTIP2 associates with the NuRD complex on the promoter of p57KIP2, a newly identified CTIP2 target gene. J Biol Chem 281, 32272–32283 (2006). 16950772 10.1074/jbc.M602776200 PMC2547407

[b17] Dehennaut V. & Leprince D. Implication of HIC1 (Hypermethylated In Cancer 1) in the DNA damage response. Bull. Cancer 96, E66–72 (2009). 19822477 10.1684/bdc.2009.0959

[b18] Carter M. G. *et al.* Mice deficient in the candidate tumor suppressor gene Hic1 exhibit developmental defects of structures affected in the Miller-Dieker syndrome. Hum. Mol. Genet. 9, 413–9 (2000). 10655551 10.1093/hmg/9.3.413

[b19] Grimm C. *et al.* Isolation and embryonic expression of the novel mouse gene Hic1, the homologue of HIC1, a candidate gene for the Miller-Dieker syndrome. Hum. Mol. Genet. 8, 697–710 (1999). 10072440 10.1093/hmg/8.4.697

[b20] Fleuriel C. *et al.* HIC1 (Hypermethylated in Cancer 1) epigenetic silencing in tumors. Int. J. Biochem. Cell Biol. 41, 26–33 (2009). 18723112 10.1016/j.biocel.2008.05.028 PMC2631403

[b21] Britschgi C. *et al.* HIC1 tumour suppressor gene is suppressed in acute myeloid leukaemia and induced during granulocytic differentiation. Br. J. Haematol. 141, 179–87 (2008). 18318772 10.1111/j.1365-2141.2008.06992.x

[b22] Rood B. R., Zhang H., Weitman D. M. & Cogen P. H. Hypermethylation of HIC-1 and 17p allelic loss in medulloblastoma. Cancer Res. 62, 3794–7 (2002). 12097291

[b23] Wales M. M. *et al.* p53 activates expression of HIC-1, a new candidate tumour suppressor gene on 17p13.3. Nat. Med. 1, 570–7 (1995). 7585125 10.1038/nm0695-570

[b24] Albagli O., Dhordain P., Deweindt C., Lecocq G. & Leprince D. The BTB/POZ domain: a new protein-protein interaction motif common to DNA- and actin-binding proteins. Cell Growth Differ. 6, 1193–8 (1995). 8519696

[b25] Deltour S., Guerardel C. & Leprince D. Recruitment of SMRT/N-CoR-mSin3A-HDAC-repressing complexes is not a general mechanism for BTB/POZ transcriptional repressors: the case of HIC-1 and gammaFBP-B. Proc. Natl. Acad. Sci. USA 96, 14831–6 (1999). 10611298 10.1073/pnas.96.26.14831 PMC24733

[b26] Chen W. Y. *et al.* Tumor Suppressor HIC1 Directly Regulates SIRT1 to Modulate p53-Dependent DNA-Damage Responses. Cell 123, 437–448 (2005). 16269335 10.1016/j.cell.2005.08.011

[b27] Deltour S., Pinte S., Guerardel C., Wasylyk B. & Leprince D. The human candidate tumor suppressor gene HIC1 recruits CtBP through a degenerate GLDLSKK motif. Mol. Cell. Biol. 22, 4890–901 (2002). 12052894 10.1128/MCB.22.13.4890-4901.2002 PMC133903

[b28] Stankovic-Valentin N. *et al.* An acetylation/deacetylation-SUMOylation switch through a phylogenetically conserved psiKXEP motif in the tumor suppressor HIC1 regulates transcriptional repression activity. Mol. Cell. Biol. 27, 2661–75 (2007). 17283066 10.1128/MCB.01098-06 PMC1899900

[b29] Pinte S. *et al.* The tumor suppressor gene HIC1 (hypermethylated in cancer 1) is a sequence-specific transcriptional repressor: definition of its consensus binding sequence and analysis of its DNA binding and repressive properties. J. Biol. Chem. 279, 38313–24 (2004). 15231840 10.1074/jbc.M401610200

[b30] Dehennaut V., Loison I., Pinte S. & Leprince D. Molecular dissection of the interaction between HIC1 and SIRT1. Biochem. Biophys. Res. Commun. 421, 384–8 (2012). 22510409 10.1016/j.bbrc.2012.04.026

[b31] Le Douce V., Cherrier T., Riclet R., Rohr O. & Schwartz C. The many lives of CTIP2: from AIDS to cancer and cardiac hypertrophy. J. Cell. Physiol. 229, 533–7 (2014). 24122342 10.1002/jcp.24490

[b32] Pagans S. *et al.* SIRT1 Regulates HIV Transcription via Tat Deacetylation. PLoS Biol 3, e41 (2005). 15719057 10.1371/journal.pbio.0030041 PMC546329

[b33] Brunet A. *et al.* Stress-dependent regulation of FOXO transcription factors by the SIRT1 deacetylase. Science 303, 2011–5 (2004). 14976264 10.1126/science.1094637

[b34] Sauve A. A. & Schramm V. L. Sir2 regulation by nicotinamide results from switching between base exchange and deacetylation chemistry. Biochemistry 42, 9249–56 (2003). 12899610 10.1021/bi034959l

[b35] Zhao X., Sternsdorf T., Bolger T. A., Evans R. M. & Yao T.-P. Regulation of MEF2 by histone deacetylase 4- and SIRT1 deacetylase-mediated lysine modifications. Mol. Cell. Biol. 25, 8456–64 (2005). 16166628 10.1128/MCB.25.19.8456-8464.2005 PMC1265742

[b36] Eilebrecht S., Wilhelm E., Benecke B.-J., Bell B. & Benecke A. G. HMGA1 directly interacts with TAR to modulate basal and Tat-dependent HIV transcription. RNA Biol. 10, 436–44 (2013). 23392246 10.4161/rna.23686 PMC3672287

[b37] Lu J. *et al.* The IFITM proteins inhibit HIV-1 infection. J. Virol. 85, 2126–37 (2011). 21177806 10.1128/JVI.01531-10 PMC3067758

[b38] Butler J. S., Dunning E. C., Murray D. W., Doran P. P. & O’Byrne J. M. HIV-1 protein induced modulation of primary human osteoblast differentiation and function via a Wnt/β-catenin-dependent mechanism. J. Orthop. Res. 31, 218–26 (2013). 23281130 10.1002/jor.22196 PMC3539237

[b39] Chen H. *et al.* CD4+ T cells from elite controllers resist HIV-1 infection by selective upregulation of p21. J Clin Invest 121, 1549–1560 (2011). 21403397 10.1172/JCI44539 PMC3069774

[b40] Dubuissez M. *et al.* Protein Kinase C-Mediated Phosphorylation of BCL11B at Serine 2 Negatively Regulates Its Interaction with NuRD Complexes during CD4+ T-Cell Activation. Mol. Cell. Biol. 36, 1881–98 (2016). 27161321 10.1128/MCB.00062-16 PMC4911745

[b41] Chi Y.-H., Semmes O. J. & Jeang K.-T. A proteomic study of TAR-RNA binding protein (TRBP)-associated factors. Cell Biosci. 1, 9 (2011). 21711701 10.1186/2045-3701-1-9 PMC3125213

[b42] Blazek D. & Peterlin B. M. Tat-SIRT1 tango. Mol. Cell 29, 539–40 (2008). 18342601 10.1016/j.molcel.2008.02.007

[b43] Kumar A., Darcis G., Van Lint C. & Herbein G. Epigenetic control of HIV-1 post integration latency: implications for therapy. Clin. Epigenetics 7, 103 (2015). 26405463 10.1186/s13148-015-0137-6 PMC4581042

[b44] Ensoli B., Cafaro A., Monini P., Marcotullio S. & Ensoli F. Challenges in HIV Vaccine Research for Treatment and Prevention. Front. Immunol. 5, 417 (2014). 25250026 10.3389/fimmu.2014.00417 PMC4157563

[b45] Bouchat S. *et al.* Histone methyltransferase inhibitors induce HIV-1 recovery in resting CD4(+) T cells from HIV-1-infected HAART-treated patients. AIDS 26, 1473–82 (2012). 22555163 10.1097/QAD.0b013e32835535f5

[b46] Darcis G. *et al.* An In-Depth Comparison of Latency-Reversing Agent Combinations in Various *In Vitro* and *Ex Vivo* HIV-1 Latency Models Identified Bryostatin-1+JQ1 and Ingenol-B+JQ1 to Potently Reactivate Viral Gene Expression. PLoS Pathog. 11, e1005063 (2015). 26225566 10.1371/journal.ppat.1005063 PMC4520688

[b47] Jiang G. *et al.* Reactivation of HIV latency by a newly modified Ingenol derivative via protein kinase Cδ-NF-κB signaling. AIDS 28, 1555–66 (2014). 24804860 10.1097/QAD.0000000000000289 PMC4922310

[b48] Jiang G. *et al.* Synergistic Reactivation of Latent HIV Expression by Ingenol-3-Angelate, PEP005, Targeted NF-kB Signaling in Combination with JQ1 Induced p-TEFb Activation. PLoS Pathog. 11, e1005066 (2015). 26225771 10.1371/journal.ppat.1005066 PMC4520526

[b49] Halper-Stromberg A. *et al.* Broadly neutralizing antibodies and viral inducers decrease rebound from HIV-1 latent reservoirs in humanized mice. Cell 158, 989–99 (2014). 25131989 10.1016/j.cell.2014.07.043 PMC4163911

[b50] Darcis G., Van Driessche B. & Van Lint C. Preclinical shock strategies to reactivate latent HIV-1: an update. Curr. Opin. HIV AIDS 11, 388–93 (2016). 27259046 10.1097/COH.0000000000000288

[b51] Kiernan R. E. *et al.* HIV-1 tat transcriptional activity is regulated by acetylation. Embo J 18, 6106–6118 (1999). 10545121 10.1093/emboj/18.21.6106 PMC1171675

[b52] Eilebrecht S. *et al.* 7SK small nuclear RNA directly affects HMGA1 function in transcription regulation. Nucleic Acids Res. 39, 2057–72 (2011). 21087998 10.1093/nar/gkq1153 PMC3064786

[b53] Eilebrecht S., Benecke B.-J. & Benecke A. 7SK snRNA-mediated, gene-specific cooperativity of HMGA1 and P-TEFb. RNA Biol. 8, 1084–93 (2011). 21957495 10.4161/rna.8.6.17015

[b54] Guarani V. *et al.* Acetylation-dependent regulation of endothelial Notch signalling by the SIRT1 deacetylase. Nature 473, 234–8 (2011). 21499261 10.1038/nature09917 PMC4598045

[b55] Marban C. *et al.* COUP-TF interacting protein 2 represses the initial phase of HIV-1 gene transcription in human microglial cells. Nucleic Acids Res 33, 2318–2331 (2005). 15849318 10.1093/nar/gki529 PMC1084325

[b56] Janabi N., Peudenier S., Heron B., Ng K. H. & Tardieu M. Establishment of human microglial cell lines after transfection of primary cultures of embryonic microglial cells with the SV40 large T antigen. Neurosci Lett 195, 105–108 (1995). 7478261 10.1016/0304-3940(94)11792-h

[b57] Brysbaert G., Pellay F.-X., Noth S. & Benecke A. Quality assessment of transcriptome data using intrinsic statistical properties. Genomics. Proteomics Bioinformatics 8, 57–71 (2010). 20451162 10.1016/S1672-0229(10)60006-X PMC5054119

[b58] Noth S., Brysbaert G., Pellay F.-X. & Benecke A. High-sensitivity transcriptome data structure and implications for analysis and biologic interpretation. Genomics. Proteomics Bioinformatics 4, 212–29 (2006). 17531797 10.1016/S1672-0229(07)60002-3 PMC5054080

[b59] Noth S. & Benecke A. Avoiding inconsistencies over time and tracking difficulties in Applied Biosystems AB1700/Panther probe-to-gene annotations. BMC Bioinformatics 6, 307 (2005). 16372901 10.1186/1471-2105-6-307 PMC1361791

[b60] Tchitchek N. *et al.* CDS: a fold-change based statistical test for concomitant identification of distinctness and similarity in gene expression analysis. Genomics. Proteomics Bioinformatics 10, 127–35 (2012). 22917185 10.1016/j.gpb.2012.06.002 PMC5054499

