# Corrigendum: HIC1 controls cellular- and HIV-1- gene transcription via interactions with CTIP2 and HMGA1

**DOI:** 10.1038/srep39569

**Published:** 2017-02-10

**Authors:** Valentin Le Douce, Faezeh Forouzanfar, Sebastian Eilebrecht, Benoit Van Driessche, Amina Ait-Ammar, Roxane Verdikt, Yoshihito Kurashige, Céline Marban, Virginie Gautier, Ermanno Candolfi, Arndt G. Benecke, Carine Van Lint, Olivier Rohr, Christian Schwartz

Scientific Reports 6: Article number: 34920; 10.1038/srep34920 published online: 10 11 2016; updated: 02 10 2017.

The affiliations for authors Valentin Le Douce, Sebastian Eilebrecht, Benoit Van Driessche, Roxane Verdikt, Yoshihito Kurashige, Céline Marban, Virginie Gautier, Arndt G. Benecke, Carine Van Lint and Olivier Rohr were incorrect in the original version of the article. The correct affiliations are listed below.

Valentin Le Douce

Université de Strasbourg, DHPI EA7292, F-67000 Strasbourg, France

Université de Strasbourg, IUT Louis Pasteur, Schiltigheim, France

UCD Centre for Research in Infectious Diseases (CRID) School of Medicine and Medical Science, University College Dublin, Ireland

Sebastian Eilebrecht

Institut des Hautes Etudes Scientifiques—Centre National de la Recherche Scientifique, 35 route de Chartres, 91440 Bures sur Yvette, France

Deutsches Krebsforschungszentrum, Im Neuenheimer Feld 242, Heidelberg 69120, Germany

Benoit Van Driessche

Université Libre de Bruxelles (ULB), Service of Molecular Virology, Institute for Molecular Biology and Medicine (IBMM), 12 rue des Profs Jeener et Brachet, 6041 Gosselies, Belgium

Roxane Verdikt

Université Libre de Bruxelles (ULB), Service of Molecular Virology, Institute for Molecular Biology and Medicine (IBMM), 12 rue des Profs Jeener et Brachet, 6041 Gosselies, Belgium

Yoshihito Kurashige

Université de Strasbourg, Inserm UMR 1121, place de l’Hôpital Strasbourg, France

Céline Marban

Université de Strasbourg, Inserm UMR 1121, place de l’Hôpital Strasbourg, France

Virginie Gautier

UCD Centre for Research in Infectious Diseases (CRID) School of Medicine and Medical Science, University College Dublin, Ireland

Arndt G. Benecke

Institut des Hautes Etudes Scientifiques—Centre National de la Recherche Scientifique, 35 route de Chartres, 91440 Bures sur Yvette, France

Université Pierre et Marie Curie, CNRS UMR 7224, 7 quai Saint Bernard, 75005 Paris, France

Carine Van Lint

Université Libre de Bruxelles (ULB), Service of Molecular Virology, Institute for Molecular Biology and Medicine (IBMM), 12 rue des Profs Jeener et Brachet, 6041 Gosselies, Belgium

Olivier Rohr

Université de Strasbourg, DHPI EA7292, F-67000 Strasbourg, France

Université de Strasbourg, IUT Louis Pasteur, Schiltigheim, France

Institut Universitaire de France, Paris, France

These errors have now been fixed in the HTML and PDF versions of the Article.

