# Supplementary material for: HIC1 controls cellular- and HIV-1- gene transcription via interactions with CTIP2 and HMGA1
Source: Sci Rep. 2016 Oct 11;6:34920. doi: 10.1038/srep34920 (PMC5057145; doi:10.1038/srep34920)
Supplement: Supplementary Table 1 [file srep34920-s1.pdf]

Table 1: Gene lists of cellular targets regulated by HIC1 and CTIP2

HIC1 controls cellular- and HIV-1- gene transcription via interactions with CTIP2 and HMGA1

Valentin Le Douce[1 2 4], Faezeh Forouzanfar[1], Sebastian Eilebrecht[8 9], Benoit Van Driessche[7] , Amina Ait-Ammar[1], Roxane Verdikt[7], Yoshihito Kurashige[5], Céline Marban[5], Ermanno Candolfi[1], Virginie Gautier[4], Arndt G. Benecke[6 9], Carine Van Lint[7#], Olivier Rohr[1 2 3#] and Christian Schwartz[1 2#]

PROBE	Entrez_Gene_ID	Symbol	Definition
ILMN_1653115	1891	ECH1	Homo sapiens enoyl Coenzyme A hydratase 1, peroxisomal (ECH1), mRNA.
ILMN_1791447	6387	CXCL12	Homo sapiens chemokine (C-X-C motif) ligand 12 (stromal cell-derived factor 1) (CXCL12), transcript variant 1, mRNA.
ILMN_1723123	2261	FGFR3	Homo sapiens fibroblast growth factor receptor 3 (achondroplasia, thanatophoric dwarfism) (FGFR3), transcript variant 2, mRNA.
ILMN_1796074	494514	C18orf56	Homo sapiens chromosome 18 open reading frame 56 (C18orf56), mRNA.
ILMN_1784602	1026	CDKN1A	Homo sapiens cyclin-dependent kinase inhibitor 1A (p21, Cip1) (CDKN1A), transcript variant 1, mRNA.
ILMN_1760593	1407	CRY1	Homo sapiens cryptochrome 1 (photolyase-like) (CRY1), mRNA.
ILMN_1811997	27246	ZNF364	Homo sapiens zinc finger protein 364 (ZNF364), mRNA.
ILMN_1743104	83759	RBM4B	Homo sapiens RNA binding motif protein 4B (RBM4B), mRNA.
ILMN_1712636	4931	NVL	Homo sapiens nuclear VCP-like (NVL), transcript variant 2, mRNA.
ILMN_1673721	9156	EXO1	Homo sapiens exonuclease 1 (EXO1), transcript variant 1, mRNA.
ILMN_1813625	7706	TRIM25	Homo sapiens tripartite motif-containing 25 (TRIM25), mRNA.
ILMN_1772677	4850	CNOT4	Homo sapiens CCR4-NOT transcription complex, subunit 4 (CNOT4), transcript variant 1, mRNA.
ILMN_1687922	6100	RP9	Homo sapiens retinitis pigmentosa 9 (autosomal dominant) (RP9), mRNA.
ILMN_1761147	2553	GABPB2	Homo sapiens GA binding protein transcription factor, beta subunit 2 (GABPB2), transcript variant gamma-2, mRNA.
ILMN_2155516	79691	QTRTD1	Homo sapiens queuine tRNA-ribosyltransferase domain containing 1 (QTRTD1), mRNA.
ILMN_1775943	84312	BRMS1L	Homo sapiens breast cancer metastasis-suppressor 1-like (BRMS1L), mRNA.

ILMN_1787509	85441	PRIC285	Homo sapiens peroxisomal proliferator-activated receptor A interacting complex 285 (PRIC285), transcript variant 2, mRNA.
ILMN_1683950	29916	SNX11	Homo sapiens sorting nexin 11 (SNX11), transcript variant 1, mRNA.
ILMN_2044617	51001	MTERFD1	Homo sapiens MTERF domain containing 1 (MTERFD1), mRNA.
ILMN_3298694	441250	TYW1B	PREDICTED: Homo sapiens misc_RNA (TYW1B), miscRNA.
ILMN_1691156	4489	MT1A	Homo sapiens metallothionein 1A (MT1A), mRNA.
ILMN_1753931	1036	CDO1	Homo sapiens cysteine dioxygenase, type I (CDO1), mRNA.
ILMN_1653712	91373	UAP1L1	Homo sapiens UDP-N-acteylglucosamine pyrophosphorylase 1-like 1 (UAP1L1), mRNA.
ILMN_2108938	23360	FNBP4	Homo sapiens formin binding protein 4 (FNBP4), mRNA.
ILMN_2080611	23590	PDSS1	Homo sapiens prenyl (decaprenyl) diphosphate synthase, subunit 1 (PDSS1), mRNA.
ILMN_2061310	55609	ZNF280C	Homo sapiens zinc finger protein 280C (ZNF280C), mRNA.
ILMN_2090802	84283	TMEM79	Homo sapiens transmembrane protein 79 (TMEM79), mRNA.
ILMN_1705241	23424	TDRD7	Homo sapiens tudor domain containing 7 (TDRD7), mRNA.
ILMN_1663532	55188	RIC8B	Homo sapiens resistance to inhibitors of cholinesterase 8 homolog B (C. elegans) (RIC8B), mRNA.
ILMN_1795678	10623	POLR3C	Homo sapiens polymerase (RNA) III (DNA directed) polypeptide C (62kD) (POLR3C), mRNA.
ILMN_1784584	60561	RINT1	Homo sapiens RAD50 interactor 1 (RINT1), mRNA.
ILMN_1664630	1111	CHEK1	Homo sapiens CHK1 checkpoint homolog (S. pombe) (CHEK1), mRNA.
ILMN_1776490	78995	C17orf53	Homo sapiens chromosome 17 open reading frame 53 (C17orf53), mRNA.
ILMN_1685661	51018	RRP15	Homo sapiens ribosomal RNA processing 15 homolog (S. cerevisiae) (RRP15), mRNA.
ILMN_1741054	8884	SLC5A6	Homo sapiens solute carrier family 5 (sodium-dependent vitamin transporter), member 6 (SLC5A6), mRNA.
ILMN_1773337	22943	DKK1	Homo sapiens dickkopf homolog 1 (Xenopus laevis) (DKK1), mRNA.
ILMN_1724145	8535	CBX4	Homo sapiens chromobox homolog 4 (Pc class homolog, Drosophila) (CBX4), mRNA.
ILMN_1662318	29080	CCDC59	Homo sapiens coiled-coil domain containing 59 (CCDC59), mRNA.
ILMN_1658504	1119	CHKA	Homo sapiens choline kinase alpha (CHKA), transcript variant 2, mRNA.
ILMN_1698533	3419	IDH3A	Homo sapiens isocitrate dehydrogenase 3 (NAD+) alpha (IDH3A), nuclear gene encoding mitochondrial protein, mRNA.
ILMN_1689652	55178	RNMTL1	Homo sapiens RNA methyltransferase like 1 (RNMTL1), mRNA.
ILMN_1745807	80021	TMEM62	Homo sapiens transmembrane protein 62 (TMEM62), mRNA.
ILMN_1736510	3344	FOXN2	Homo sapiens forkhead box N2 (FOXN2), mRNA.
ILMN_1754272	64785	GINS3	Homo sapiens GINS complex subunit 3 (Psf3 homolog) (GINS3), mRNA.
ILMN_1707199	91833	WDR20	Homo sapiens WD repeat domain 20 (WDR20), transcript variant 1, mRNA.
ILMN_1712347	644422	LOC644422	PREDICTED: Homo sapiens misc_RNA (LOC644422), miscRNA.

ILMN_1797031	79663	HSPBAP1	Homo sapiens HSPB (heat shock 27kDa) associated protein 1 (HSPBAP1), mRNA.
ILMN_1665554	55290	BRF2	Homo sapiens BRF2, subunit of RNA polymerase III transcription initiation factor, BRF1-like (BRF2), mRNA.
ILMN_1815733	1983	EIF5	Homo sapiens eukaryotic translation initiation factor 5 (EIF5), transcript variant 1, mRNA.
ILMN_1681008	10668	CGRRF1	Homo sapiens cell growth regulator with ring finger domain 1 (CGRRF1), mRNA.
ILMN_2115154	11097	NUPL2	Homo sapiens nucleoporin like 2 (NUPL2), mRNA.
ILMN_1721391	533	ATP6V0B	Homo sapiens ATPase, H+ transporting, lysosomal 21kDa, V0 subunit b (ATP6V0B), transcript variant 1, mRNA.
ILMN_2187727	64318	NOC3L	Homo sapiens nucleolar complex associated 3 homolog (S. cerevisiae) (NOC3L), mRNA.
ILMN_1663667	80308	FLAD1	Homo sapiens FAD1 flavin adenine dinucleotide synthetase homolog (S. cerevisiae) (FLAD1), transcript variant 2, mRNA.
ILMN_1652409	55812	SPATA7	Homo sapiens spermatogenesis associated 7 (SPATA7), transcript variant 2, mRNA.
ILMN_2279873	123720	WHAMM	Homo sapiens WAS protein homolog associated with actin, golgi membranes and microtubules (WHAMM), mRNA.
ILMN_3305938	6446	SGK1	Homo sapiens serum/glucocorticoid regulated kinase 1 (SGK1), transcript variant 1, mRNA.
ILMN_1767892	11266	DUSP12	Homo sapiens dual specificity phosphatase 12 (DUSP12), mRNA.
ILMN_2067852	7779	SLC30A1	Homo sapiens solute carrier family 30 (zinc transporter), member 1 (SLC30A1), mRNA.
ILMN_1773868	8233	U2AF1L2	Homo sapiens U2(RNU2) small nuclear RNA auxiliary factor 1-like 2 (U2AF1L2), mRNA.
ILMN_1759513	390	RND3	Homo sapiens Rho family GTPase 3 (RND3), mRNA.
ILMN_1677305	5817	PVR	Homo sapiens poliovirus receptor (PVR), mRNA.
ILMN_1781102	79609	C14orf138	Homo sapiens chromosome 14 open reading frame 138 (C14orf138), transcript variant 2, mRNA.
ILMN_1674243	7037	TFRC	Homo sapiens transferrin receptor (p90, CD71) (TFRC), mRNA.
ILMN_1702487	6446	SGK	Homo sapiens serum/glucocorticoid regulated kinase (SGK), mRNA.
ILMN_2049727	51526	C20orf111	Homo sapiens chromosome 20 open reading frame 111 (C20orf111), mRNA.
ILMN_1701131	79074	C2orf49	Homo sapiens chromosome 2 open reading frame 49 (C2orf49), mRNA.
ILMN_1769245	11010	GLIPR1	Homo sapiens GLI pathogenesis-related 1 (GLIPR1), mRNA.
ILMN_1711005	993	CDC25A	Homo sapiens cell division cycle 25 homolog A (S. pombe) (CDC25A), transcript variant 1, mRNA.
ILMN_1676984	1649	DDIT3	Homo sapiens DNA-damage-inducible transcript 3 (DDIT3), mRNA.
ILMN_1725612	10762	NUP50	Homo sapiens nucleoporin 50kDa (NUP50), transcript variant 2, mRNA.
ILMN_2317658	6520	SLC3A2	Homo sapiens solute carrier family 3 (activators of dibasic and neutral amino acid transport), member 2 (SLC3A2), transcript variant 1, mRNA.
ILMN_1794017	29950	SERTAD1	Homo sapiens SERTA domain containing 1 (SERTAD1), mRNA.
ILMN_1667825	197259	MLKL	Homo sapiens mixed lineage kinase domain-like (MLKL), mRNA.
ILMN_2221564	55646	LYAR	Homo sapiens Ly1 antibody reactive homolog (mouse) (LYAR), mRNA.
ILMN_1781721	64794	DDX31	Homo sapiens DEAD (Asp-Glu-Ala-Asp) box polypeptide 31 (DDX31), transcript variant 1, mRNA.

ILMN_1657893	10587	TXNRD2	Homo sapiens thioredoxin reductase 2 (TXNRD2), nuclear gene encoding mitochondrial protein, mRNA.
ILMN_1726456	6520	SLC3A2	Homo sapiens solute carrier family 3 (activators of dibasic and neutral amino acid transport), member 2 (SLC3A2), transcript variant 6, mRNA.
ILMN_1774589	55721	IQCC	Homo sapiens IQ motif containing C (IQCC), mRNA.
ILMN_1748591	4953	ODC1	Homo sapiens ornithine decarboxylase 1 (ODC1), mRNA.
ILMN_1731720	81572	PDRG1	Homo sapiens p53 and DNA-damage regulated 1 (PDRG1), mRNA.
ILMN_1666553	60386	SLC25A19	Homo sapiens solute carrier family 25 (mitochondrial thiamine pyrophosphate carrier), member 19 (SLC25A19), nuclear gene encoding mitochondrial prote
ILMN_1733703	55687	TRMU	Homo sapiens tRNA 5-methylaminomethyl-2-thiouridylate methyltransferase (TRMU), nuclear gene encoding mitochondrial protein, transcript variant 1, m
ILMN_1783276	91624	NEXN	Homo sapiens nexilin (F actin binding protein) (NEXN), mRNA.
ILMN_1729179	79029	SPATA5L1	Homo sapiens spermatogenesis associated 5-like 1 (SPATA5L1), mRNA.
ILMN_1667162	4824	NKX3-1	Homo sapiens NK3 homeobox 1 (NKX3-1), mRNA.
ILMN_2117716	8227	SFRS17A	Homo sapiens splicing factor, arginine/serine-rich 17A (SFRS17A), transcript variant 1, mRNA.
ILMN_1770228	3885	KRT34	Homo sapiens keratin 34 (KRT34), mRNA.
ILMN_1674983	387841	LOC387841	PREDICTED: Homo sapiens similar to ribosomal protein L13a, transcript variant 2 (LOC387841), mRNA.
ILMN_1670752	22889	KIAA0907	Homo sapiens KIAA0907 (KIAA0907), mRNA.
ILMN_1785852	64859	OBFC2A	Homo sapiens oligonucleotide/oligosaccharide-binding fold containing 2A (OBFC2A), mRNA.
ILMN_2225577	134359	C5orf37	Homo sapiens chromosome 5 open reading frame 37 (C5orf37), mRNA.
ILMN_1791728	114789	SLC25A25	Homo sapiens solute carrier family 25 (mitochondrial carrier; phosphate carrier), member 25 (SLC25A25), nuclear gene encoding mitochondrial protein, tra
ILMN_1807136	729559	LOC729559	PREDICTED: Homo sapiens similar to ciliary rootlet coiled-coil, rootletin (LOC729559), mRNA.
ILMN_1787511	80745	THUMPD2	Homo sapiens THUMP domain containing 2 (THUMPD2), mRNA.
ILMN_1659936	23645	PPP1R15A	Homo sapiens protein phosphatase 1, regulatory (inhibitor) subunit 15A (PPP1R15A), mRNA.
ILMN_1682775	1906	EDN1	Homo sapiens endothelin 1 (EDN1), mRNA.
ILMN_1693242	162979	ZNF296	Homo sapiens zinc finger protein 296 (ZNF296), mRNA.
ILMN_1898124			Homo sapiens cDNA FLJ38860 fis, clone MESAN2011977
ILMN_3248773	285958	C7orf40	Homo sapiens chromosome 7 open reading frame 40 (C7orf40), non-coding RNA.
