# Supplementary material for: HIC1 controls cellular- and HIV-1- gene transcription via interactions with CTIP2 and HMGA1
Source: Sci Rep. 2016 Oct 11;6:34920. doi: 10.1038/srep34920 (PMC5057145; doi:10.1038/srep34920)
Supplement: Supplementary Table 2 [file srep34920-s2.pdf]

Table 2: Gene lists of cellular targets regulated by HIC1 and HMGA1

HIC1 controls cellular- and HIV-1- gene transcription via interactions with CTIP2 and HMGA1

Valentin Le Douce[1 2 4], Faezeh Forouzanfar[1], Sebastian Eilebrecht[8 9], Benoit Van Driessche[7] , Amina Ait-Ammar[1], Roxane Verdikt[7], Yoshihito Kurashige[5], Céline Marban[5], Ermanno Candolfi[1], Virginie Gautier[4], Arndt G. Benecke[6 9], Carine Van Lint[7#], Olivier Rohr[1 2 3#] and Christian Schwartz[1 2#]

PROBE	Entrez_Gene_ID	Symbol	Definition
ILMN_1653115	1891	ECH1	Homo sapiens enoyl Coenzyme A hydratase 1, peroxisomal (ECH1), mRNA.
ILMN_1784602	1026	CDKN1A	Homo sapiens cyclin-dependent kinase inhibitor 1A (p21, Cip1) (CDKN1A), transcript variant 1, mRNA.
ILMN_2083946	7039	TGFA	Homo sapiens transforming growth factor, alpha (TGFA), mRNA.
ILMN_1684183	5883	RAD9A	Homo sapiens RAD9 homolog A (S. pombe) (RAD9A), mRNA.
ILMN_1787509	85441	PRIC285	Homo sapiens peroxisomal proliferator-activated receptor A interacting complex 285 (PRIC285), transcript variant 2, mRNA.
ILMN_1745282	5891	RAGE	Homo sapiens renal tumor antigen (RAGE), mRNA.
ILMN_1682015	51083	GAL	Homo sapiens galanin prepropeptide (GAL), mRNA.
ILMN_1791679	92737	DNER	Homo sapiens delta/notch-like EGF repeat containing (DNER), mRNA.
ILMN_1725387	114801	TMEM200A	Homo sapiens transmembrane protein 200A (TMEM200A), mRNA.
ILMN_2357134	8877	SPHK1	Homo sapiens sphingosine kinase 1 (SPHK1), transcript variant 1, mRNA.
ILMN_2090802	84283	TMEM79	Homo sapiens transmembrane protein 79 (TMEM79), mRNA.
ILMN_1809364	4908	NTF3	Homo sapiens neurotrophin 3 (NTF3), mRNA.
ILMN_1712347	644422	LOC644422	PREDICTED: Homo sapiens misc_RNA (LOC644422), miscRNA.
ILMN_1784948	90853	SPOCD1	Homo sapiens SPOC domain containing 1 (SPOCD1), mRNA.
ILMN_1768754	29990	PILRB	Homo sapiens paired immunoglobin-like type 2 receptor beta (PILRB), transcript variant 1, mRNA.
ILMN_1688480	595	CCND1	Homo sapiens cyclin D1 (CCND1), mRNA.
ILMN_1770338	4071	TM4SF1	Homo sapiens transmembrane 4 L six family member 1 (TM4SF1), mRNA.
ILMN_1794017	29950	SERTAD1	Homo sapiens SERTA domain containing 1 (SERTAD1), mRNA.
ILMN_1770228	3885	KRT34	Homo sapiens keratin 34 (KRT34), mRNA.
ILMN_1682775	1906	EDN1	Homo sapiens endothelin 1 (EDN1), mRNA.
